# 3D-printed devices for continuous-flow organic chemistry

**DOI:** 10.3762/bjoc.9.109

**Published:** 2013-05-16

**Authors:** Vincenza Dragone, Victor Sans, Mali H Rosnes, Philip J Kitson, Leroy Cronin

**Affiliations:** 1 School of Chemistry, University of Glasgow, University Avenue, Glasgow G12 8QQ, UK. Web: http://www.croninlab.com

**Keywords:** 3D printing, flow chemistry, flow IR, in-line analysis, imine reduction, imine synthesis, millifluidics, reactionware

## Abstract

We present a study in which the versatility of 3D-printing is combined with the processing advantages of flow chemistry for the synthesis of organic compounds. Robust and inexpensive 3D-printed reactionware devices are easily connected using standard fittings resulting in complex, custom-made flow systems, including multiple reactors in a series with in-line, real-time analysis using an ATR-IR flow cell. As a proof of concept, we utilized two types of organic reactions, imine syntheses and imine reductions, to show how different reactor configurations and substrates give different products.

## Introduction

The use of flow chemistry and 3D-printing technology is expanding in the field of organic synthesis [1–5]. The application of continuous-flow systems is frequently found in chemistry, and is beginning to have a significant impact on the way molecules are made [1–3]; on the other hand the application of 3D-printing technology in synthetic chemistry still has many aspects that can be investigated. The benefits resulting from the utilization of 3D-printing techniques to create bespoke reactionware for synthetic chemistry have recently been reported [4–5].

3D printing consists of the fabrication of three-dimensional physical objects from a digital model [6]. The 3D printer takes the virtual design from computer-aided design (CAD) software and reproduces it layer-by-layer until the physical definition of the layers gives the designed object. The significant advantage of this technique is that the architecture can be concisely controlled. 3D printing allows chemists to build devices with high precision, including complex geometries and intricate internal structures such as channels with well-defined size dimensions. Furthermore, understanding the kinetics of the processes can allow the (re-)designing of the reactionware, allowing us to combine additional kinetic knowledge with reactor designs. Moreover, the additive manufacturing process of the devices takes a short time and results in a cheap procedure for the fabrication of fluidic devices [7]. All this is important in chemistry, and in particular for the realization of micro- and millifluidic devices.

Microfluidic devices compatible with a wide range of organic solvents and reagents are usually made of silicon or glass, which requires specialized manufacturing techniques and are expensive to fabricate [8]. There is growing interest in the use of polymers that can be employed to fabricate devices in a rapid and inexpensive fashion [9]. One of the most commonly employed polymers is poly(dimethylsiloxane) (PMDS), due to its low cost and the possibility of rapid prototyping. Nevertheless, it is not suitable for carrying out organic reactions as it can absorb the reactants and will swell in most nonaqueous solvents [8]. 3D-printing technology offers the possibility of employing polypropylene (PP), a thermopolymer that is inert in a range of organic solvents and organic compounds, cheaper than PMDS, and compatible with the available 3D printers.

Herein, we demonstrate the versatility and convenience of using 3D-printed reactors for the synthesis of organic compounds, using flow techniques with an in-line ATR-IR flow cell to monitor the reactions in real time. There are several examples of different techniques used for real-time analyses in the literature, such as UV–vis [4–5,10–11], IR [5,10,12–14], and even NMR spectroscopy [15–17]. The use of in-line spectroscopy allows for the monitoring of reaction steps that include unstable compounds or hazardous species [18]. Further, the use of such techniques may also be used to obtain quantitative information about reaction progress and to rapidly optimize the reaction conditions “on the fly”.

First, an in-house designed and 3D-printed reactionware device was employed for the synthesis of imines from the reaction of a range of aldehydes and primary amines. Secondly, two reactors were connected in series to first perform an imine synthesis and then subsequently an imine reduction, with this second setup showing the potential for using the 3D-printed devices as reliable tools in multistep synthesis. This showed that the simplicity of designing and building flow reactors employing 3D-printing techniques allows for an easy and convenient integration of devices in a flow setup. Therefore it represents a very attractive way to design and build new continuous-flow rigs for organic synthesis.

## Results and Discussion

### Experimental setup

The 3D-printed flow reactors used to carry out the organic syntheses were designed by using a 3D CAD software package (Autodesk123D^®^), which is freely distributed and produces files that can be converted to the correct format read by the 3DTouch^TM^ printer. This 3D printer heats a thermopolymer through the extruder, depositing the material in a layer-by-layer fashion, converting the design into the desired 3D reactionware.

The thermoplastic employed to fabricate the devices presented herein is PP, selected to print robust, inexpensive and chemically inert devices. Comparing PP with other common and accessible thermoplastics, which have been used in 3D printing before, such as polylactic acid (PLA) and polyacrylates, in PP we can find the required characteristics to perform a chemical reaction: thermostability up to 150 °C, high chemical inertia, and low cost. PLA is widely used in medicinal chemistry because of its biocompatibility; however, from a chemical point of view its use is limited to a few solvents and organic compounds, and to preserve its integrity it can only be used up to temperatures of 60–66 °C [19]. Polyacrylates consist of a vast group of polymers with different physical and chemical properties; however their chemical compatibility is low. In fact they are not generally recommended for exposure to alcohol, glycols, alkalis, brake fluids, or to chlorinated or aromatic hydrocarbons [20]. Therefore, PP was the plastic of choice for the device fabrication.

**Figure 1:**
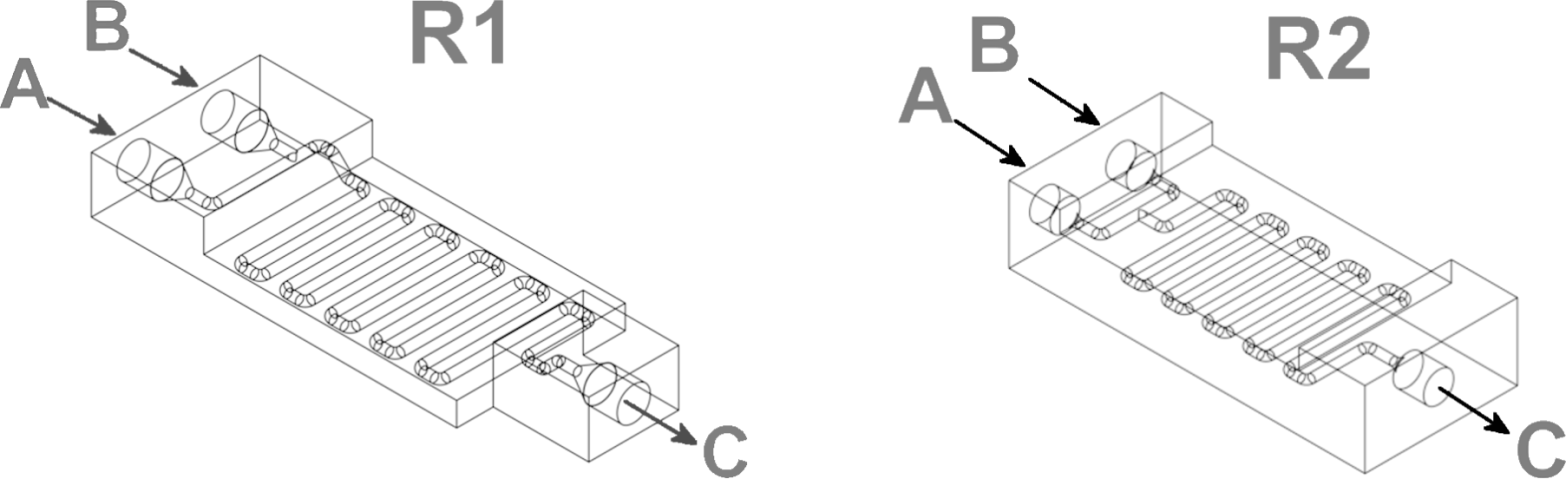
Schematic representation of the 3D-printed reactionware devices employed in this work showing the internal channels. Both have two inputs (A and B) and one output (C). The main difference consists in the length of the inlets/outlets: the dimension of the inlets/outlets in R1 is 3 mm and in R2 it is 6 mm where the latter is designed to match the size of standard check-valves.

The shape of the 3D-printed reactionware devices used herein (Figure 1) was chosen in order to combine a short design and print time with the robustness required for a flow system.

Each device has two inlets, followed by a mixing point, a length of reactor to ensure a controlled residence time (which is given by dividing the reactor volume by the total flow rate), and one outlet. The approximate volume of the first reactor (R1, see Figure 1, left) is ca. 0.4 mL and was employed in the imine syntheses, while the second reactor (R2, see Figure 1, right) has a volume of ca. 0.35 mL and was employed connected to another R2 for the imine reduction processes. All the characteristics of the devices are summarized in Table 1.

**Table 1:** 3D-printed reactionware device characteristics.

Entry	Characteristics	R1	R2

1	printing time (min)	248	367
2	PP mass (g)	24.01	33.74
3	dimensions (mm)	30 × 80.2 × 10	70 × 30 × 15
4	internal diameter (mm)	1.5	1.5
5	theoretical volume^a^ (mL)	0.54	0.51
6	reactor volume	0.4	0.35

^a^The theoretical internal volumes of the devices are higher than the measured volumes. This is due to the printing process, where the internal channel diameter is always slightly smaller than the designed one.

**Figure 2:**
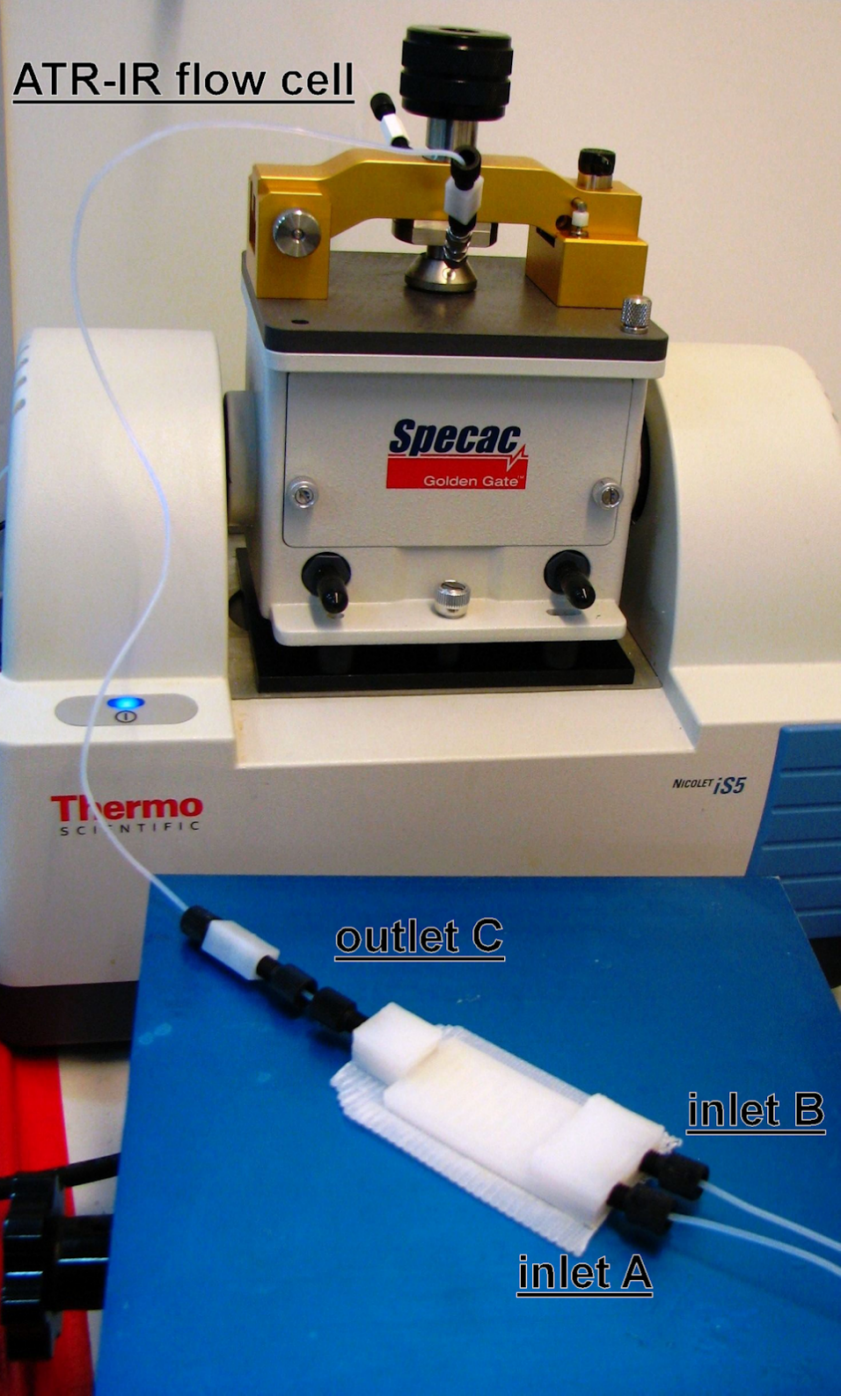
Flow system setup, where a R1 is connected to the syringe pumps and the ATR-IR flow cell with standard connectors.

The 3D-printed devices were integrated in the flow systems using 1.58 mm outer diameter (OD) polytetrafluoroethylene (PTFE) tubing, with an internal diameter of 0.5 mm and standard connectors made of polyfluoroelastomer (FPM) and polyether ether ketone (PEEK). PEEK is a harder plastic than PP and, thus, allowed the screwing of the standard connectors into the softer PP inlets/outlets of the devices, resulting in a tight seal to the device. The screw connectors increase the chemical tolerance of the 3D-printed reactor as well as its chemical compatibility, compared to our previous devices [5]. The connectors at the device inlets were equipped with check valves (made of PEEK with a Chemraz^®^ O-ring, which is compatible with organic solvents and compounds) to prevent potential backflow issues. The reactor inlets were connected to the syringe pumps containing the starting material solutions, whilst the outlets were connected to the in-line ATR-IR flow cell (see Figure 2). These improvements are a considerable step forward compared to our previous report on 3D printing fluidics [5], as they facilitate the integration of the devices, increase the chemical compatibility, improve the range of pressure that can be handled by the system, and enable the easy configuration for the use of ancillary equipment.

### Device 1: Imine formation

**Figure 3:**
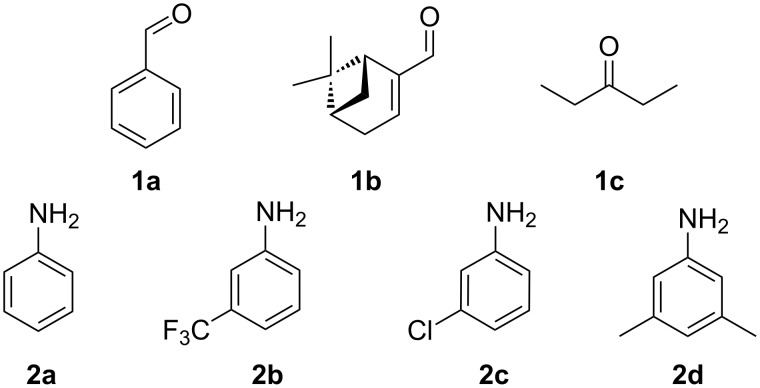
Carbonyl compounds and primary amines used in the syntheses reported in this work. Carbonyl compounds: benzaldehyde (**1a**); R-(−)-myrtenal (**1b**); 3-pentanone (**1c**). Aniline derivatives: aniline (**2a**); 3-(trifluoromethyl)aniline (**2b**); 3-chloroaniline (**2c**); 3,5-dimethylaniline (**2d**).

Here we show the 3D-printed device as a millifluidic reactor for the synthesis of imines under flow conditions. We monitored the reaction progress with the help of an in-line ATR-IR flow cell, which is a very useful technique for the monitoring of organic reactions under flow conditions [10,21–26]. The flow setup used for these syntheses consists of two syringe pumps, each of them connected to one of the inlets of the 3D-printed reactionware device R1. The syringe pumps were filled with the starting materials with a carbonyl compound (**1a**–**c**) being placed in syringe pump no. 1 and with a primary amine (**2a**–**d**) being placed in syringe pump no. 2 (Figure 3).

The experiments were conducted using 2 M methanolic solutions of the different substrates. This is convenient from a processing point of view, since high concentrations favor increased reaction kinetics [26] whilst minimizing the amount of waste generated during the downstream work-up [27]. The reactor output was connected with a length of tubing with a volume 0.1 mL to the IR flow cell. Hence, the total flow reactor volume (*V*_R_) was 0.5 mL. The syntheses of the imines were monitored by an in-line ATR-IR flow cell and were conducted at a total flow rate of 0.25 mL min^−1^, where two equimolar methanolic solutions of **1** and **2** were flowed into R1 at the same flow rate. The residence time was calculated as the time taken for the solutions to go from the mixing point inside the 3D-printed reactor to the analytical device, thus taking into account the subsequent pieces of tubing employed, and resulted to be 2 minutes. The choice of a short residence time is to allow for a more reliable comparison of the imines synthesized and also to avoid the formation of the Michael addition adduct [28] (the thermodynamic compound) in the reaction between compounds **1b** and **2a**.

For the first experiment, we studied the reaction of benzaldehyde (**1a**) with the aniline derivatives **2a**–**d** (Figure 3), to synthesize the *N*-benzylideneanilines **3a**–**d** (see Table 2). The different substituents on the amine compounds have an electronic effect on the reactive center, thus influencing the observed conversion, i.e., an electron-donating group (EDG) in the meta-position of the aniline ring gives a higher percentage conversion than does an electron-withdrawing group (EWG) [28]. In fact, Table 2 shows that the conversion of benzaldehyde (**1a**) to imine **3a** (Table 2, entry 1; obtained by reacting **1a** with **2a**), is higher than with the conversion of **1a** to imine **3b** (Table 2, entry 2; obtained by reacting **1a** with **2b**). The conversion of **1a** to imine **3c** (Table 2, entry 3; reaction of **1a** with **2c**), is the same as the formation of **3a**, whilst the formation of **3d** (reaction of **1a** with **2d**) has the highest conversion %.

**Table 2:** Conversion of benzaldehyde (**1a**) into imines **3a**–**d**.

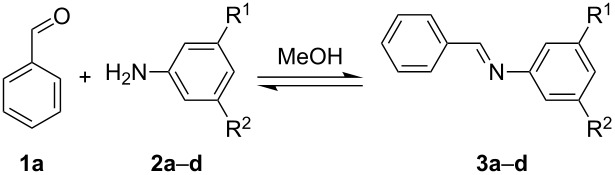		

Entry	Product	Conversion (%)

1	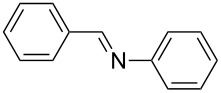 **3a**	96
2	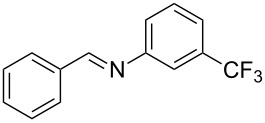 **3b**	85
3	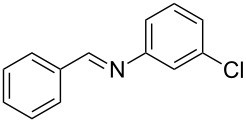 **3c**	96
4	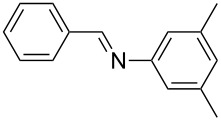 **3d**	99

**Figure 4:**
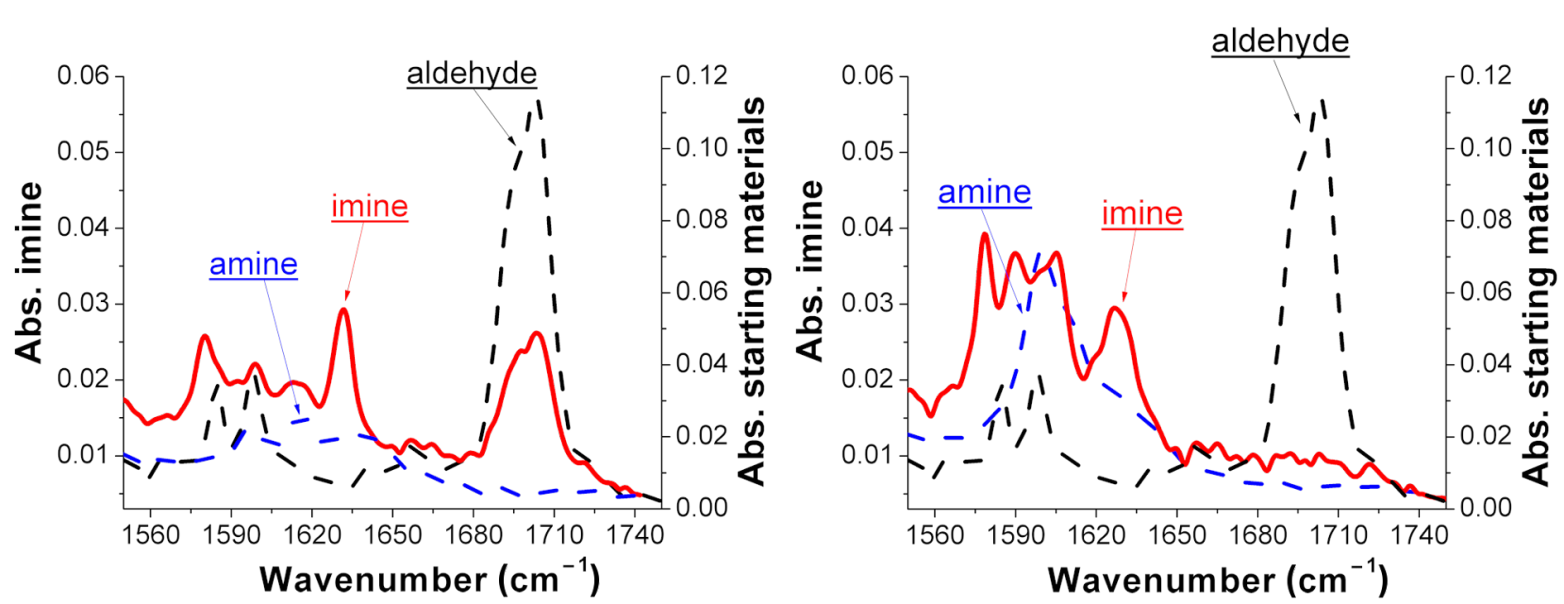
ATR-IR spectra of the synthesis of compounds **3b** (on the left) and **3d** (on the right). The spectrum on the left shows the reaction that does not go to completion due to the EWG substituent on the meta-position of the primary amine **2b** (see Supporting Information File 1).

Figure 4 shows the effect of the EWG and EDG substituents of a phenyl ring through the IR spectra of compounds **3b** (on the left) and **3d** (on the right). In both graphs the imine spectrum (in red) is compared with the spectrum of the starting materials (dash line): the aldehyde peak of benzaldehyde (**1a**) at 1704 cm^−1^ (in black) disappears when it reacts with compound **2d** (Figure 4, on the left), while it is still present when combined with compound **2b** (Figure 4, on the right). ^1^H NMR spectroscopy was used to confirm the conversion rate of **1a** to the *N*-benzylideneaniline derivatives **3a**–**d**.

**Figure 5:**
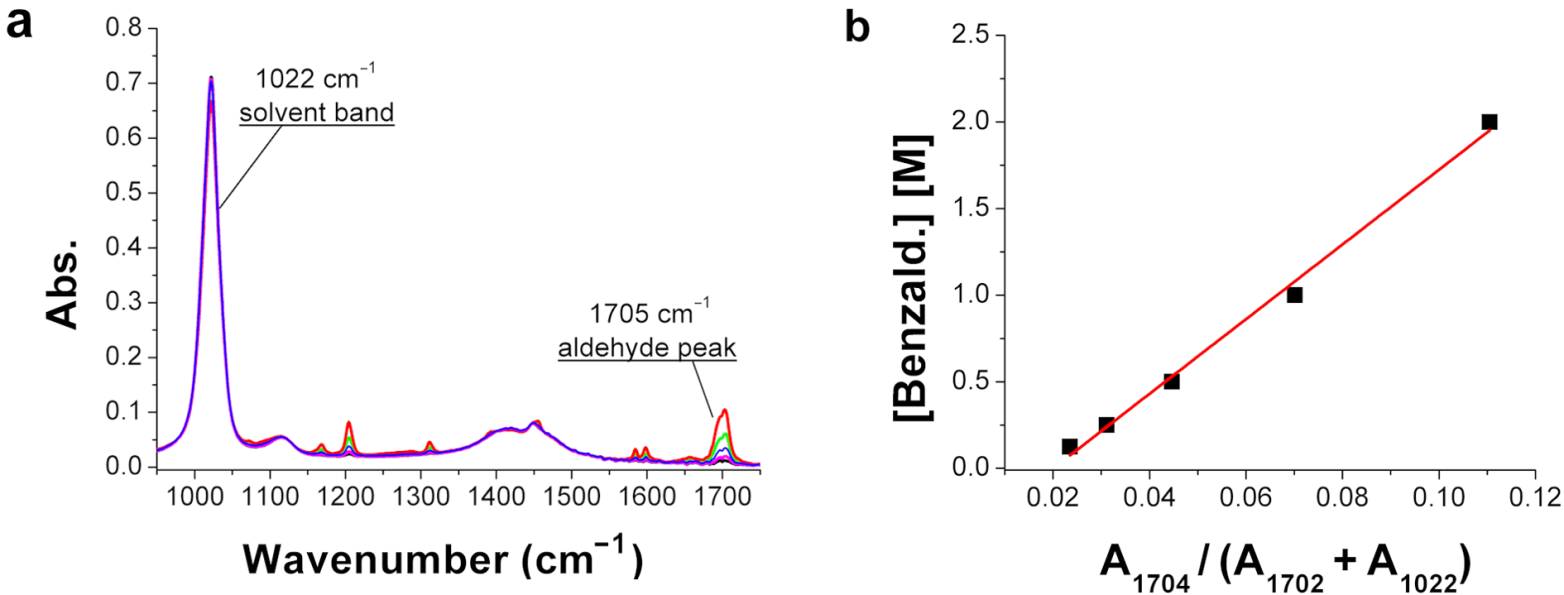
**(**a) IR spectra of benzaldehyde at different concentrations. The solvent peak at 1022 cm^−1^ remains constant while the aldehyde peak at 1704 cm^−1^ increases with the concentration of benzaldehyde. (b) Calibration curve of the different molar concentrations of benzaldehyde is shown. Equation 1: [benzaldehyde] = −0.432 + 21.56 × A_1704_ / (A_1022_ + A_1704_) and the *R*^2^ = 0.993.

To calculate the conversion of the benzaldehyde (**1a**) into the imines **3a**–**d** when combined with the amines **2a**–**d**, a calibration of the IR spectra of benzaldehyde at known concentrations was obtained. The different concentrations of the substrates used for the IR analysis do not significantly affect the intensity of the area of the solvent band at 1022 cm^−1^ (A_1022_). Hence, it is possible to use the solvent peaks to normalize the different spectra, allowing for comparison of the results. From this data a calibration curve can be obtained dividing the area of the benzaldehyde band at 1704 cm^−1^ (A_1704_) by A_1022_, calculated for five different molar concentrations of the methanolic solutions of benzaldehyde. We used 2 M, 1 M, 0.5 M, 0.25 M and 0.125 M methanolic solutions of benzaldehyde, and the relative areas were calculated using the corrected solvent-band area (A_s_*) and adding A_1704_ to it, in order to minimize the slight change of A_1022_ with the concentration of the benzaldehyde (Figure 5).

**Figure 6:**
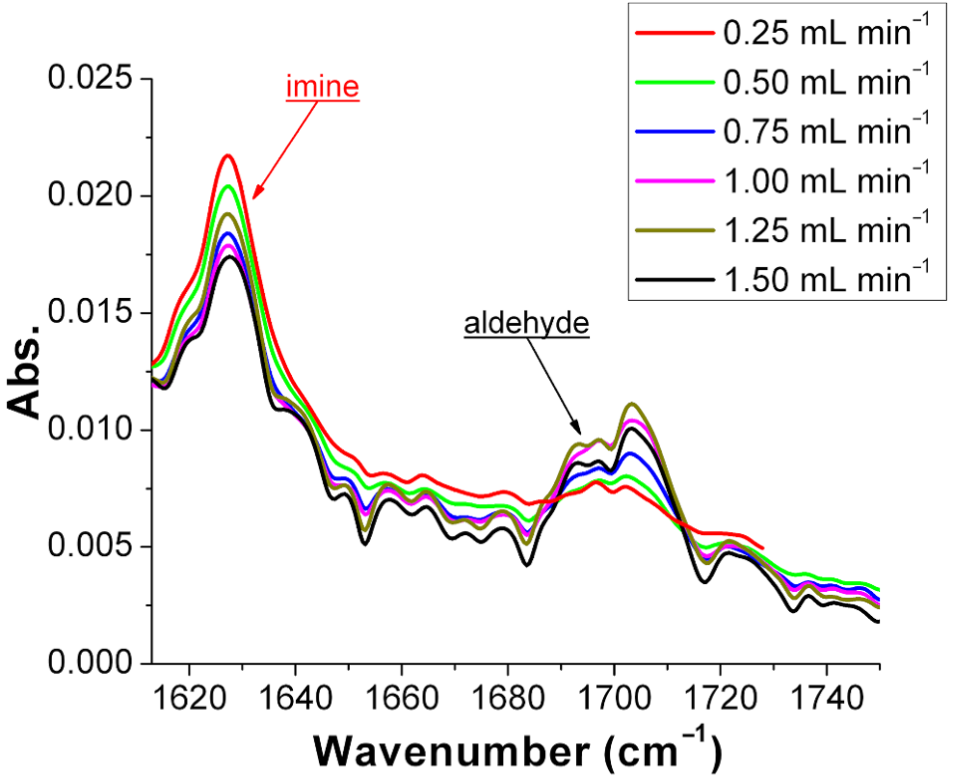
Comparison of the IR spectra of imine **3a**, derived from benzaldehyde (**1a**) and aniline (**2a**), synthesized at different flow rates. The conversion of **3a** at different flow rates was calculated using the equation of the calibration curve (see Figure 4), and for a flow rate of 0.25 mL min^−1^ was 97% and at a flow rate of 1.5 mL min^−1^, 94%.

Different flow rates were assayed to elucidate the effect of the reaction time. To synthesize imine **3a**, equimolar amounts of benzaldehyde (**1a**) and aniline (**2a**) were mixed in ratio 1:1 (v/v) at different flow rates in the range 0.25–1.5 mL min^−1^. The reported spectra are focused in the region of the IR spectra where the conversion of aldehyde **1a** to imine **3a** can be followed (see Figure 6). Following the red spectra (synthesis of **3a** with the shortest residence time) it can be seen that the imine band at 1627 cm^−1^ is more intense compared to the one in black (synthesis of **3a** with the highest residence time). The observed conversion range found was between 94% and 97%. Under the studied conditions, very high conversions have been obtained with residence times as low as 20 seconds.

Further imine syntheses in-flow were conducted with the 3D-printed millifluidic reactor R1 and monitored with the in-line ATR-IR (Table 3).

**Table 3:** Conversion of carbonyl compounds **1b** and **1c** with aniline (**2a**) into imines **3e** and **3f**.

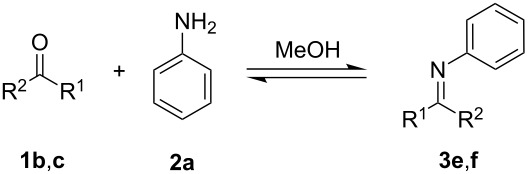		

Entry	Product	Conversion (%)

1	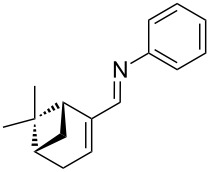 **3e**	94
2	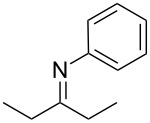 **3f**	–

The results of these reactions are summarized in Table 3 where it can be seen that the reaction between aniline (**2a**) and R-(−)-myrtenal (**1b**) readily takes place to give imine **3e** (Table 3, entry 1), whilst no product can be observed under these conditions for the reaction of **2a** with 3-pentanone (**1c**), due to the lower reactivity of the latter. For details, see the IR spectrum of compound **3f** in section 5 of Supporting Information File 1. ^1^H NMR spectra were used to calculate the conversion rate of aldehyde **1b** into imine **3e**.

### Device 2: Imine reduction

**Figure 7:**
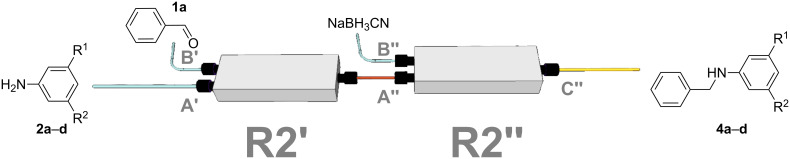
Representation of the setup for the two-step flow reaction employed in this work. The first reactor (R2’) is used to synthesize the imines under previously optimized conditions. The product is then directly introduced into the next reactor (R2”) and mixed with the reducing agent to produce the secondary amine.

To further prove the reliability of the 3D-printed devices as flow reactors, we decided to connect one reactor to the other and perform a two steps flow reaction in an automated way. To this end, we employed two R2 reactionware devices connected in series (Figure 7), to monitor the formation of the final product using the in-line ATR-IR flow cell. We ran the imine synthesis in the first of the two reactors (R2’), and once formed we subsequently reduced it in the second reactor (R2”). R2’ was connected to the syringe pumps containing the starting materials (compounds **1a** and **2a**–**d**) for the imine synthesis as previously described (but with a longer residence time than described above, to ensure a complete conversion of the substrates), before imines **3a**–**d** were directly introduced to R2” for the subsequent reduction.

The reduction of imines is a strategy to synthesize functionalized secondary amines [23–24], although only a few examples of reductions in microfluidic devices have been reported in the literature [5,23–25]. The condensation reactions were conducted using a 2 M solution of benzaldehyde (**1a**) in MeOH as before, which was pumped through inlet B’ into reactor R2’ at 0.0125 mL min^−1^ and mixed with a 2 M solution of the aniline derivatives **2a**–**d** in MeOH introduced through inlet A’ at the same flow rate, keeping the aldehyde/amine ratio (1:1) (v/v) as described for the imine synthesis in R1. We selected this low flow rate to obtain a sufficient residence time (*t*_R_ = 14 min) for a full conversion of **1a** into imines **3a**–**d**. Reactor R2’ was connected to the inlet A” of a second device (R2”) where the freshly formed imine was mixed with the reducing agent, cyanoborohydride (NaBH_3_CN) in MeOH (1 M), introduced through inlet B”, and the two equimolar solutions were pumped through R2” at the same flow rate. The molar and volumetric ratios hydride/imine were kept constant (1:1) to produce the corresponding amines with a residence time of 7 min. The reducing agent was selected because it is mild but effective, and it prevents the undesired formation of bubbles or problems related to over-reduction, which could be expected in this range of concentrations when using conventional reducing agents, such as NaBH_4_. Using this methodology, imines **3a**–**d** were reduced affording the corresponding secondary amines **4a**–**d** (Table 4).

**Table 4:** Table of the compounds used to study the imine reduction.

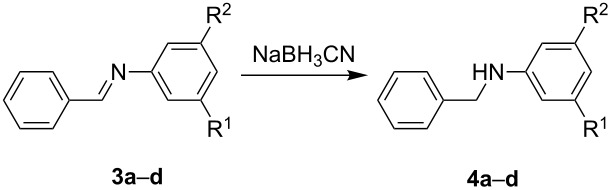		

Entry	Product **4**	Yield (%)

1	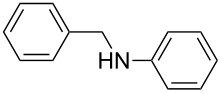 **4a**	78
2	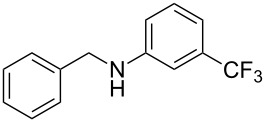 **4b**	99
3	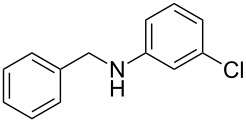 **4c**	96
4	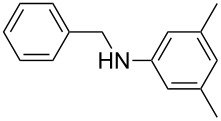 **4d**	97

^1^H NMR spectroscopy and MS spectrometry confirmed the presence of the amines. ^1^H NMR spectra were used to calculate the conversion rate of the amines **4a**–**d**.

**Figure 8:**
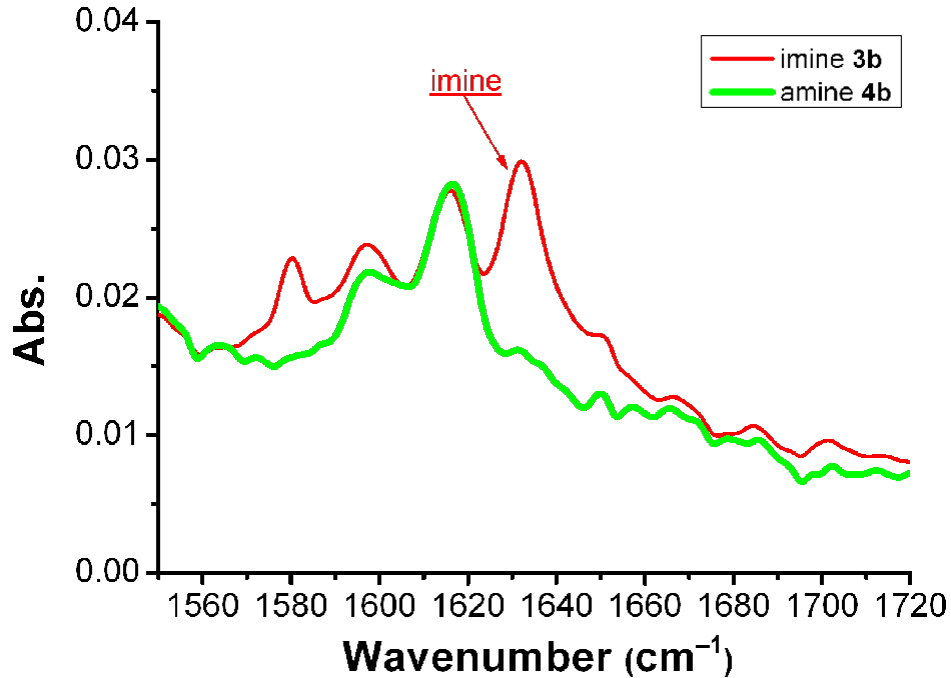
Example of an ATR-IR graph in which an imine spectrum is compared with the reduced imine spectrum.

The reactions were followed by monitoring the absence of the imine and aldehyde bands in the in-line ATR-IR flow cell, focusing the attention on the region of the IR spectrum between 1720 cm^−1^ and 1550 cm^−1^, where the disappearance of the imine band (around 1630 cm^−1^) can be observed. Figure 8 shows the spectra of imine **3b** (red) and its corresponding reduced product, compound **4b** (green) as an example; in the red spectrum a complete conversion of the aldehyde into imine **3b** can be observed (due to the absence of the aldehyde peak at 1704 cm^−1^), and in the green spectrum the imine peak at 1632 cm^−1^ has completely disappeared.

In addition to the IR analysis, compounds **4a**–**d** were collected and analyzed by mass spectrometry (MS), HPLC and ^1^H NMR spectroscopy. In all the studied cases, the analytical data confirmed full conversion of the substrates into the corresponding amines.

## Conclusion

We have demonstrated that it is possible to integrate 3D-printed reactionware devices into a flow system, which highlights the great versatility and modularity of 3D-printed reaction devices. The possibility of connecting the reactors using standard fittings allows for better seals and facilitates the reuse of the devices, compared to our previously published procedures [5]. Further, the versatility of the 3D-printed reactionware has been demonstrated by studying and optimizing the residence time to synthesize a range of imines and secondary amines and to monitor the reactions in real time using in-line IR spectroscopy.

These robust, inexpensive and chemically inert 3D-printed reactors have proven suitable vessels for single-step as well as multistep reactions in flow. The chemical and thermal stability of PP makes this generation of custom built flow reactors suitable for the investigation of more complex chemistry. Therefore the next step will be to design and print reactionware devices tailored to selected chemistry, such as by increasing the inlets/outlets numbers, adapting the channel size to the different stages of a reaction, and including reservoir chambers, etc.

We strongly believe that the ease of combining robust and cheap devices with other instruments in the laboratory can lead us to build new reactionware for the faster optimization of chemical processes as well as opening the potential for the discovery and implementation of array chemistry. We are currently investigating the effect of the device architecture on the reaction performed by using 3D-printed reactors made of PP, testing their robustness and chemical inertia in different environments, and designing new geometries to further develop the 3D printing technology and the 3D-printed reactionware, as well as the development of a range of universal chemical modules.

## Supporting Information

File 1 3D printing materials and method, experimental and characterization of compounds.
