# Supplementary material for: 3D-printed devices for continuous-flow organic chemistry
Source: Beilstein J Org Chem. 2013 May 16;9:951–9. doi: 10.3762/bjoc.9.109 (PMC3678713; doi:10.3762/bjoc.9.109)
Supplement: File 1 — 3D printing materials and method, experimental and characterization of compounds. [file Beilstein_J_Org_Chem-09-951-s001.pdf]

Supporting Information

for

3D-printed devices for continuous-flow organic chemistry

Vincenza Dragone, Victor Sans, Mali H. Rosnes, Philip J. Kitson, Leroy Cronin*

Address: School of Chemistry, University of Glasgow, University Avenue, Glasgow G12 8QQ, UK. Web: http://www.croninlab.com

Email: Leroy Cronin - Lee.Cronin@glasgow.ac.uk

* Corresponding author

3D printing materials and method, experimental and characterization of compounds

Table of contents

1. 3D printing materials and methods S2
    1.1 Design software S2
    1.2 3D printer S2
2. Experimental S3
    2.1 Materials and methods S3
    2.2 Device setups S3
    2.3 IR spectroscopy S3
    2.4 Mass spectrometry S3
    2.5 NMR spectroscopy S3
    2.6 HPLC S4
3. Imine synthesis S4
    3.1 Synthesis and characterization S4
    3.2 ^{1}H NMR spectrum of compound **3e** S6
4. Imine reduction S7
    4.1 Secondary amine synthesis flow setup S7
    4.2 Synthesis and characterization S7
5. IR spectra S9

1 3D printing materials and methods

1.1 Design software

The 3D-printed reactionware devices used in this work were designed on the freely distributed 3D CAD software Autodesk123D® (http://www.123dapp.com/) although any 3D modeling/CAD software with the ability to export models into an .STL file format would suffice for this, and there are a number of suitable alternative free/open source candidates available on the internet. The device designs were exported as .STL files (available from the authors), which was then interpreted by Bits from Bytes Axon 2 software, which produces a 3D printer instruction file (.bfb file) which was subsequently transferred to the 3DTouch™ 3D printer. The printing was conducted in a layer-by-layer fashion by the 3DTouch™ printer, and the devices were printed using polypropylene (PP), and were subsequently fitted with standard PTFE 1/16” OD tubing and connectors.

1.2 3D printer

The 3D printer used in this work has a print tolerance of +/- 0.2 mm and the Z resolution (i.e. the height of each layer of deposited thermopolymer) of the printer was 0.125 mm (+/- 0.06 mm), allowing the printing of channels which are approximately circular in cross section and with a channel diameter of approximately 1.5 mm. The printing of reactor R1 took about 4 hours, and 6 hours for R2. Before any reactions were initiated the devices were tested using a dye solution of rhodamine B in methanol, to confirm that the devices had been printed accurately and that there were no leaks in the channel systems.

2 Experimental

2.1 Materials and methods

All chemical reagents and solvents were purchased from Sigma–Aldrich and used without further purification.

2.2 Device setups

All solutions were pumped by means of C-3000 syringe pumps from Tricontinent equipped with 1 mL syringes. An in-house developed LabVIEW application was employed to program the pumps to deliver the desired flow rates and to control the IR spectroscopy.

2.3 IR spectroscopy

IR spectra were collected in-line employing a Nicolet IS-5 from Thermo Scientific and a ZnSe Golden Gate ATR from Specac equipped with a flow cell. The resolution was set at 4 cm^{-1} and 16–80 scans were recorded.

2.4 Mass spectrometry

The spectra were recorded using a JEOL JMS 700 (EI/CI). The observed ca. *m*/*z* values are listed.

2.5 NMR spectroscopy

All NMR data were recorded on a Bruker Advance 400 MHz, in deuterated MeOH from Goss Scientific, at T = 300 K. All chemical shifts are given in ppm. The peaks are denoted s = singlet, d = doublet, t = triplet, dt = doublet of triplet, and m = multiplet.

2.6 HPLC

High performance liquid chromatography (HPLC) was performed using a 150 × 2.00 mm, 3 µm Phenomenex column on a Agilent 1100 Series equipped with UV detector with a source light of 254 wavelength at 25 °C. A mixture of acetonitrile (HPLC gradient grade) and a buffer, made of sodium acetate, tuned to pH 3.72, was used in a ratio of 95:5 as eluent. 10 µL of each sample (1 M) was diluted in 1 mL of acetonitrile and 1 µL of this solution was injected at a flow rate of 0.5 mL min^{-1}.

3 Imine formation

3.1 Synthesis and characterization

A 2 M methanolic solution of carbonyl compounds (**1a**–**c**) was mixed with a 2 M methanolic solution of primary amines (**2a**–**d**) at the same flow rate (0.125 mL min^{-1}), using the 3D-printed reactionware device R1, with a total reactor volume of 0.5 mL (0.4 mL + 0.1 mL), a residence time of about 2 min and a total flow rate being 0.25 mL min^{-1}. The outlet of the reactor was connected to the flow-cell of a Golden Gate ATR-IR. The products were analyzed in-line by flow ATR-IR, using an aliquot of the reaction mixture for MS and HPLC analyses, and after evaporation of the solvent, for ^{1}H NMR spectroscopy.

***N*-Benzylideneaniline (3a):** This imine was synthesized by reacting compounds **1a** and **2a**. ^{1}H NMR: (400 MHz, $CDCl_3$) (δ, ppm) 8.56 (s, 1H), 8.00–7.97 (m, 2H), 7.58–7.59 (m, 3H), 7.54–7.44 (m, 3H), 7.36–7.31 (m, 2H); MS (EI^+): calcd. for $C_{13}H_{11}N$ (M^+) *m/z* 181.08, found 181.15; HPLC: t_R = 1.22 min; IR: 1703 cm^{-1} peak from C=O moiety disappeared, 1627 cm^{-1} peak from C=N-C moiety appeared.

***N*-Benzylidene-3-(trifluoromethyl)aniline (3b):** This imine was synthesized by reacting compounds **1a** and **2b**. ^{1}H NMR: (400 MHz, $CDCl_3$) (δ, ppm) 8.48 (s, 1H),

7.96–7.93 (m, 2H), 7.54–7.52 (m, 5H), 7.47 (s, 1H), 7.40 (m, 1H); MS (EI^+): calcd. for $C_{14}H_{10}F_3N$ (M^+) *m/z* 249.08, found 249.13; HPLC: t_R = 1.25 min; IR: 1703 cm^{-1} peak from C=O moiety disappeared, 1632 cm^{-1} peak from C=N-C moiety appeared.

***N*-Benzylidene-3-chloroaniline (3c):** This imine was synthesized by reacting compounds **1a** and **2c**. ^{1}H NMR: (400 MHz, $CDCl_3$ (δ, ppm) 8.32 (s, 1H), 7.95–7.95 (m, 2H), 7.60–7.55 (m, 3H), 7.44–7.39 (m, 1H), 7.31–7.28 (m, 2H), 7.20-7.17 (m, 1H); MS (CI^+): calcd. for $C_{13}H_{10}ClN$ (M^+) *m/z* 215.05, found 215.12; HPLC: t_R = 1.30 min; IR: 1703 cm^{-1} peak from C=O moiety disappeared, 1631 cm^{-1} peak from C=N-C moiety appeared.

***N*-Benzyliden-3,5-dimethylaniline (3d):** This imine was synthesized by reacting compounds **1a** and **2d**. ^{1}H NMR: (400 MHz, $CDCl_3$) (δ, ppm) 8.47 (s, 1H), 7.93–7.90 (m, 2H), 7.50–7.48 (m, 3H), 6.91 (s, 1H), 6.87 (s, 2H), 2.38 (s, 6H); MS (EI^+): calcd. for $C_{15}H_{15}N$ (M^+) *m*/*z* 209.12, found 209.19; HPLC: t_R = 1.44 min; IR: 1703 cm^{-1} peak from C=O moiety disappeared, 1627 cm^{-1} peak from C=N-C moiety appeared.

(6,6-Dimethyl-bicyclo[3.1.1]hept-2-en-2-ylmethylene)aniline (3e): This imine was synthesized by reacting compounds **1b** and **2a**. ^{1}H NMR: (400 MHz, $CDCl_3$) (δ, ppm) 8.03 (s, 1H), 7.38–7.34 (m, 2H), 7.21–7.16 (m, 1H), 7.14–7.11 (m, 2H), 6.28–6.27 (m, 1H), 3.20 (td, 1H, J_d = 1.3 Hz, J_t = 5.6 Hz), 2.58–2.56 (m, 4H), 2.25–2.20 (m, 1H), 1.41 (s, 3H), 1.06 (d, 1H, J = 9.3 Hz), 0.87 (s, 3H); MS (EI^+): calcd. for $C_{16}H_{19}N$ (M^+) *m/z* 225.15, found 225.2; IR: 1673 cm^{-1} peak from C=O moiety disappeared, 1586 cm^{-1} peak from C=N-C moiety appeared.

3.2 ^{1}H NMR spectrum of compound 3e

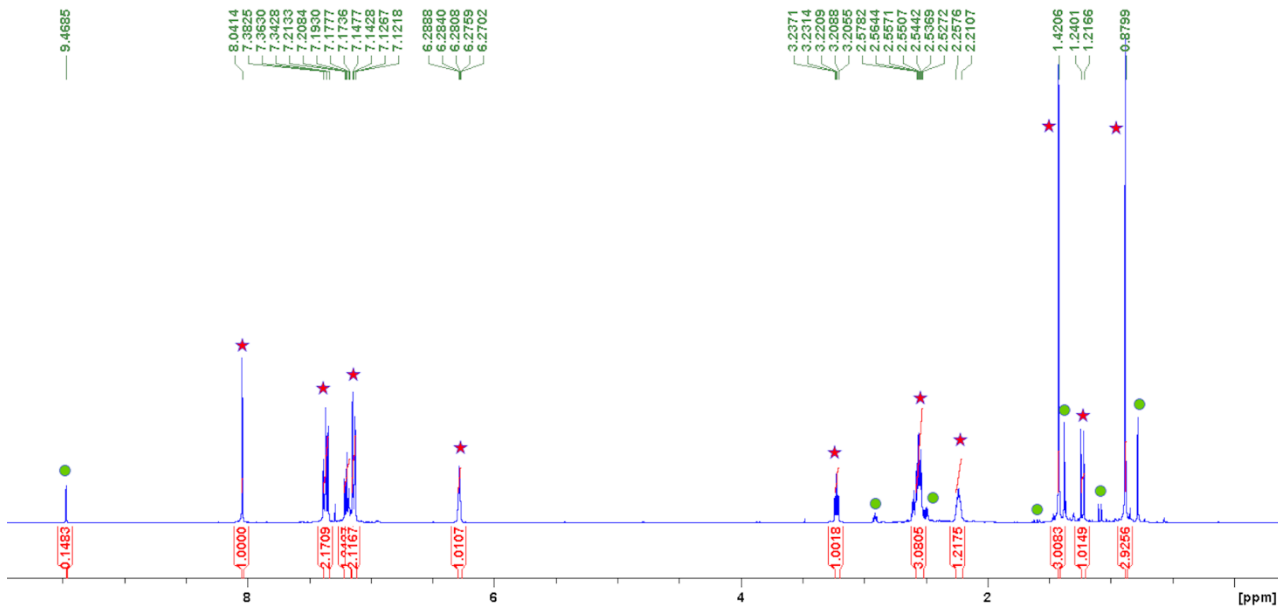

Figure S1: ^{1}H NMR (400 MHz, 298K, $CDCl_3$) spectrum of imine **3e**. The signals of imine **3e** (indicated with a star) and aldehyde **2e** (indicated with a circle) are both present, as results of this equilibrium reaction. Therefore it is possible to calculate the conversion of **2e** to **3e** using the integral values of the aldehyde-proton signal at 9.47 ppm and the one of the imine-proton signal at 8.04 ppm. The conversion of **2e** to **3e** is about 87%.

4 Imine reduction

4.1 Secondary amine synthesis flow setup

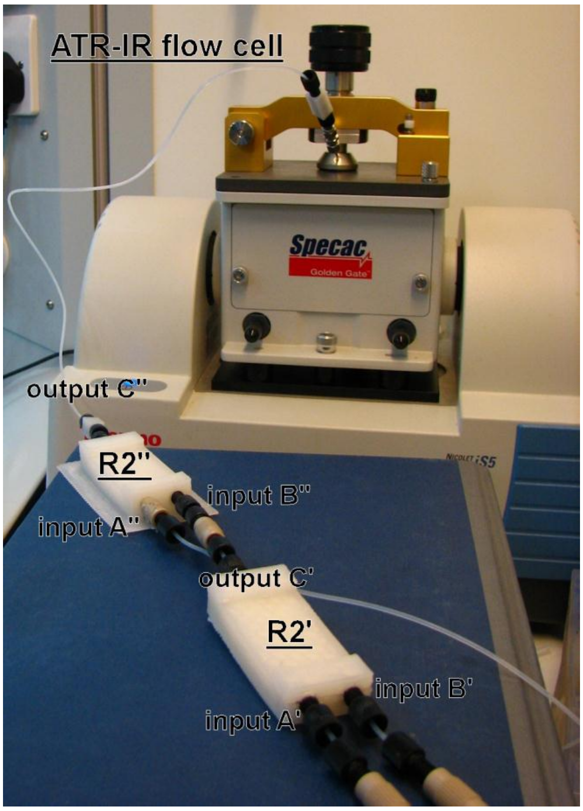

Figure S2: Flow setup for the imine reduction. The inlets A’ and B’ of the first reactor are connected to two syringe pumps that can be filled with aldehyde and amine solutions. The outlet C’ is connected to one of the inlet (B”) of the second reactor; the other inlet is connected to a third syringe pump that can be filled with the reducing agent. The output of the second device (C”) is connected to the ATR-IR flow cell through a 0.1 mL PTFE tube equipped with screw fittings.

4.2 Synthesis and characterization

Two 2 M methanolic solutions of a primary amine (**2a–d**) and benzaldehyde (**1a**) were pumped into reactor R2’, at the same flow rate of 0.0125 mL min^{-1}, and with the aldehyde/amine ratio (1:1) (1 mL:1 mL). In this second reactor R2” each of the resulting compounds **3a–d** was mixed using a flow rate of 0.025 mL min^{-1} with a 1 M

methanolic solution of cyanoborohydride, at the same flow rate, and using a molar and volumetric ratio hydride/imine of (1:1) (1 mL:1 mL) with the total flow rate of 0.05 mL min^{-1} and the residence time in R2" of 7 minutes, a secondary amine (**4a-d**) was synthesized. The samples were characterized by in-line flow ATR-IR, using an aliquot of the reaction mixture for MS and HPLC analyses, and after evaporation of the solvent, for ^{1}H NMR spectroscopy.

Benzyl-phenylamine (4a): This amine was obtained by reducing the compound **3a**. **^{1}H NMR**: (400 MHz, $CDCl_3$) (δ, ppm) 7.46–7.40 (m, 7H), 6.79–6.69 (m, 3H) 4.38 (s, 2H), 4.36 (s, 1H); MS (EI^+): calcd. for $C_{13}H_{13}N$ (M^+) *m/z* 183.1, found 183.2; HPLC: t_R = 1.18 min; IR: 1627 cm^{-1} peak from C=N moiety disappeared, 1603 cm^{-1} peak from CH_2NH-Ph moiety appeared.

Benzyl-(3-trifluoromethyl-phenyl)-amine (4b): This amine was obtained by reducing the compound **3b**. ^{1}H NMR: (400 MHz, $CDCl_3$) (*δ*, ppm) 7.45–7.40 (m, 5H), 6.99-6.97 (m, 2H), 6.92 (s, 1H), 6.85–6.82 (m, 2H) 4.42 (s, 2H), 4.25 (s, 1H); MS (EI^+): calcd. for $C_{14}H_{12}$ F_3N (M^+) *m/z* 251.09, found 251.1; HPLC: t_R = 1.15 min; IR: 1632 cm^{-1} peak from C=N moiety disappeared, 1617 cm^{-1} peak from CH_2NH-Ph moiety appeared.

Benzyl-(3-chloro-phenyl)-amine (4c): This amine was obtained by reducing the compound **3c**. ^{1}H NMR: (400 MHz, $CDCl_3$) (δ, ppm) 7.42–7.38 (m, 5H), 7.11 (t, *J* = 7.8 Hz, 3H), 6.73 (s, 1H), 6.72–6.70 (dd, *J* = 7.8 Hz, *J = 2.3* Hz, 1H), 6.56–6.53 (dd, *J* = 7.8 Hz, *J = 2.3* Hz, 1H) 4.36 (s, 2H), 3.92 (s, 1H); MS (CI^+): calcd. for $C_{13}H_{13}ClN$ (MH^+) *m/z* 218.07, found 218.1; HPLC: t_R = 1.11 min; IR: 1631 cm^{-1} peak from C=N moiety disappeared, 1600 cm^{-1} peak from CH_2NH-Ph moiety appeared.

Benzyl-(3,5-dimethylphenyl)-amine (4d): This amine was obtained by reducing the compound **3d**. ^{1}H NMR: (400 MHz, $CDCl_3$) (δ, ppm) 7.45–7.36 (m, 5H), 6.43 (s, 1H), 6.34 (s, 2H), 4.34 (s, 2H), 4.03 (s, 1H) 2.27 (s, 6H); MS (EI^+): calcd. for $C_{15}H_{17}N$ (M^+)

m/z 211.14, found 211.2; HPLC: t_R = 1.23 min; IR: 1627 cm^{-1} peak from C=N moiety disappeared, 1603 cm^{-1} peak from CH_2NH-Ph moiety appeared.

5 IR spectra

Herein are the IR spectra measured in-line with an ATR-IR flow cell during the reaction between the different aldehydes (compounds **1a** and **1b**) and amines (compounds **2a**–**d**), to obtain the imines **3a**–**f** reported along with the reduction of the compounds **3a**–**d** into secondary amines **4a**–**d**. In each spectra the starting materials are reported in dash lines, and the color legend is the following: the carbonyl compounds **1a**–**c** are shown in black, the aniline derivatives **2a**–**d** in blue, and the mixture of reaction measured in-line with the ATR-IR flow cell during the imine synthesis and the imine reduction, corresponding to the compounds **3a**–**f** and **4a**–**d** are respectively in red and in green.

Compound 3a.

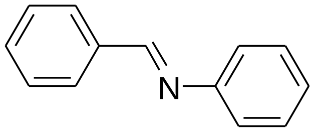

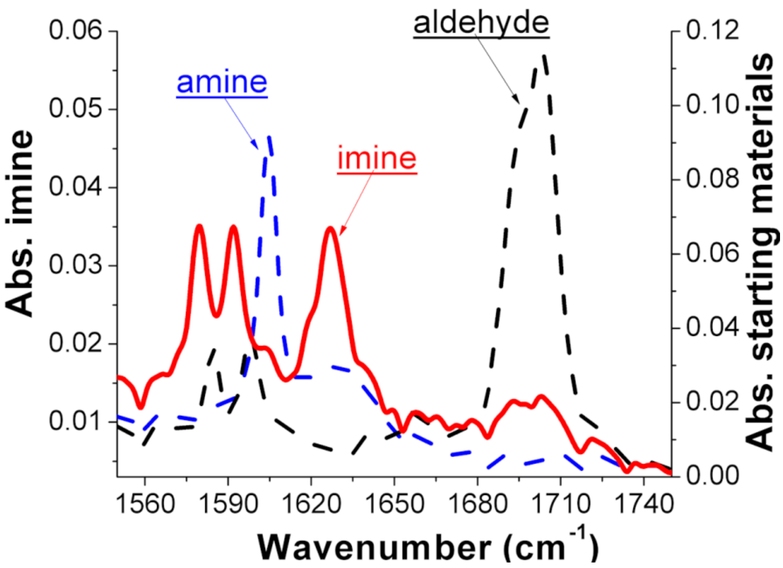

Compound 3b.

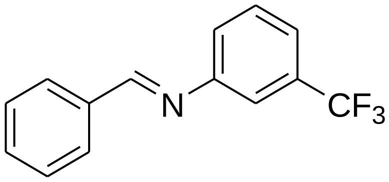

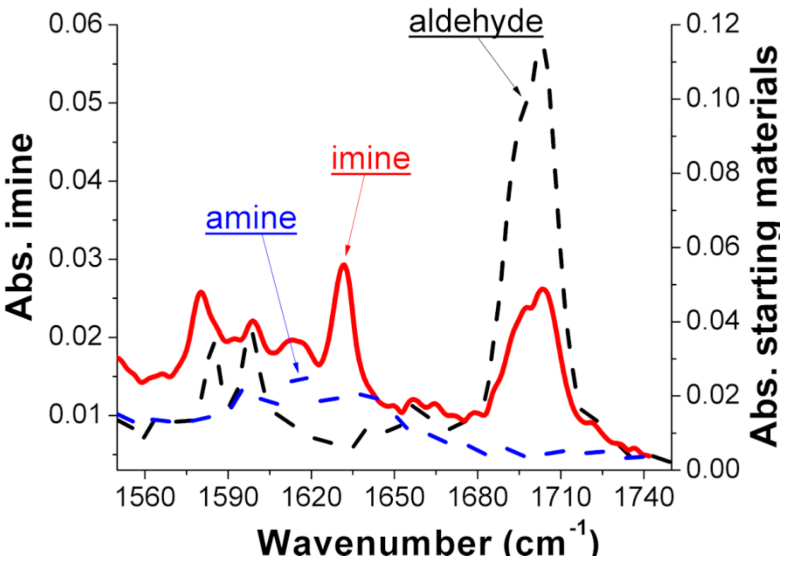

Compound 3c.

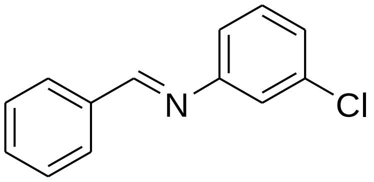

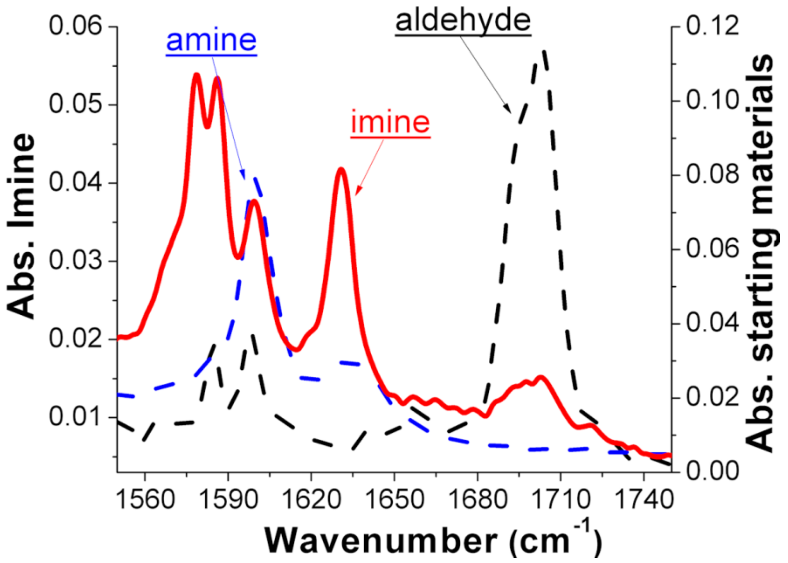

Compound 3d.

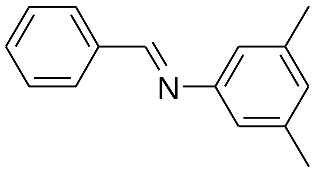

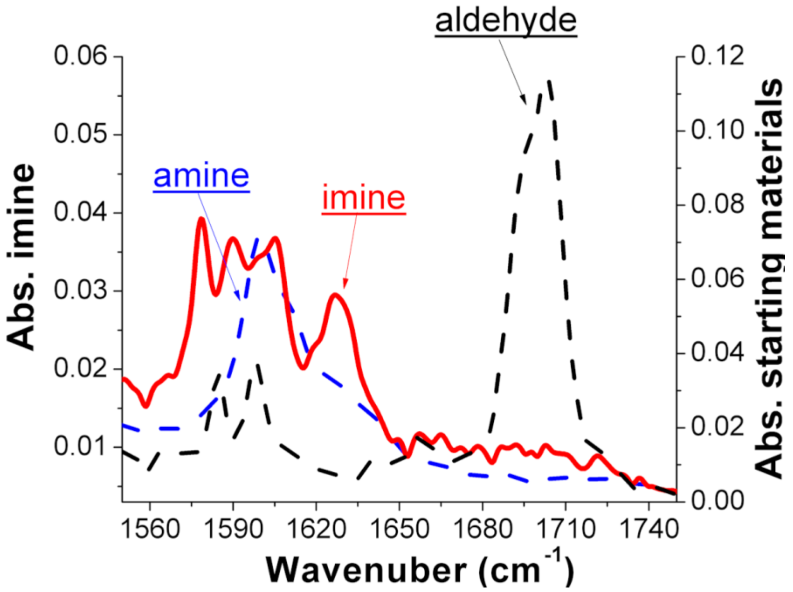

Compound 3e.

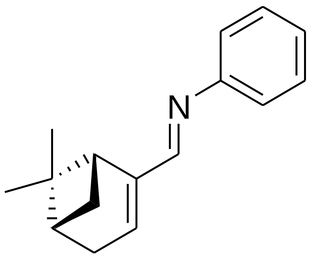

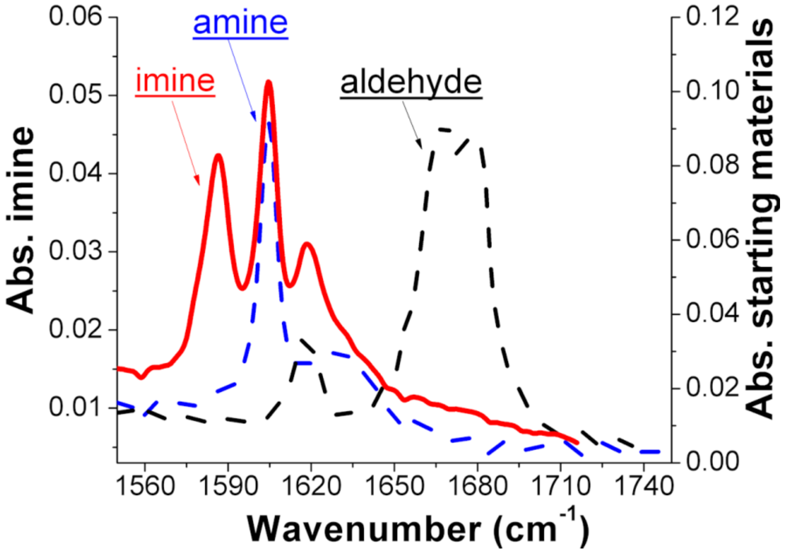

Compound 3f.

Aniline reactivity with pentanone: Amine is in blue, the carbonyl compound in black, and in red is the result of the reaction.

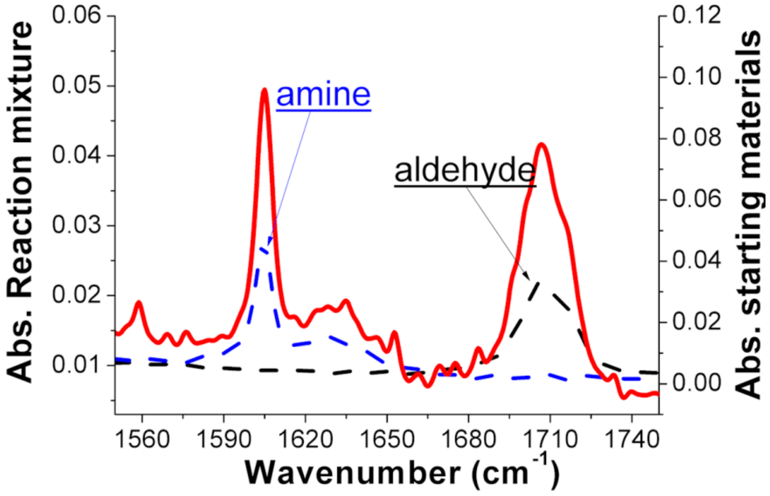

Compound 4a.

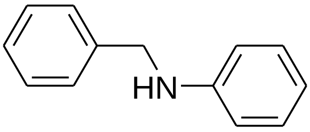

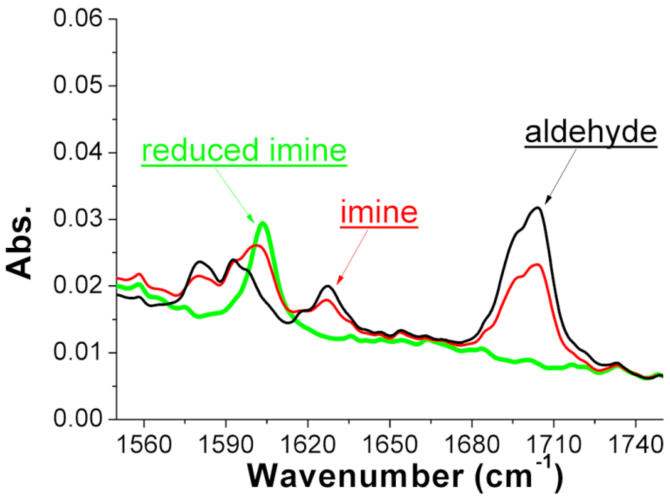

Compound 4b.

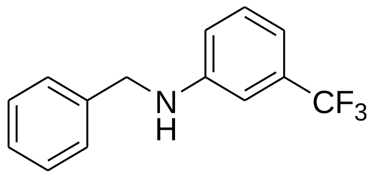

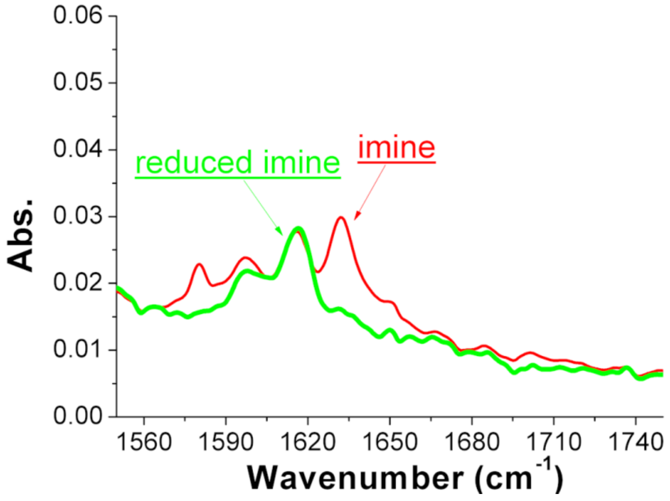

Compound 4c.

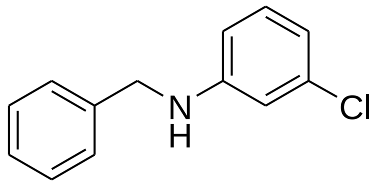

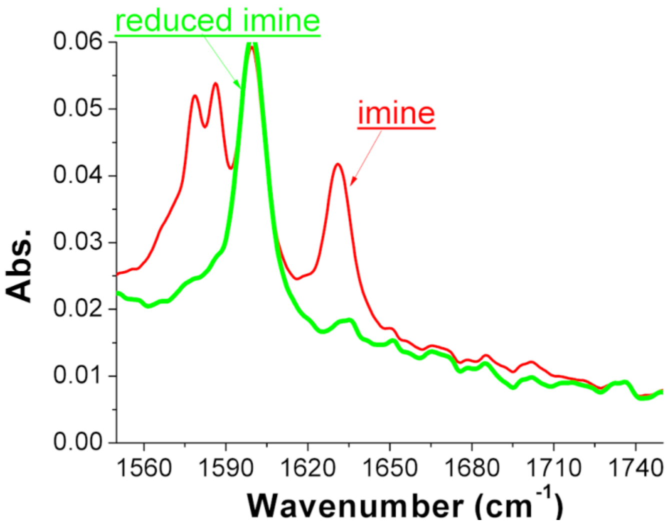

Compound 4d.

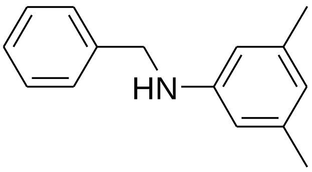

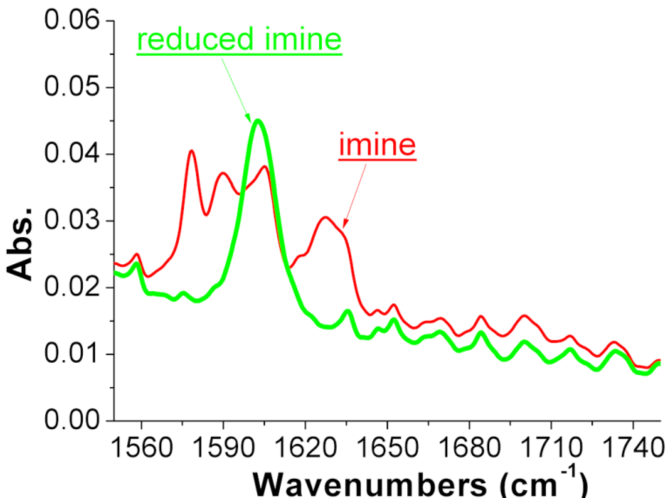

reduced imine
imine
Abs.
0.06
0.05
0.04
0.03
0.02
0.01
0.00
1560
1590
1620
1650
1680
1710
1740
Wavenumbers (cm-1)
